# Psychological Health and Risk Factors of College Students within the Context of the COVID-19 Pandemic: A Cross-Sectional Study

**DOI:** 10.3390/bs13100796

**Published:** 2023-09-26

**Authors:** Feilong Lv, Rui Zhu, Xiaorong Hou, Laihao Fang, Yanzhi Wang, Zhiyin Du, Xiaoni Zhong, Jiaxiu Liu

**Affiliations:** 1 College of Medical Informatics, Chongqing Medical University, Chongqing 400016, China; 2 Medical Data Science Academy, Chongqing Medical University, Chongqing 400016, China; 3 First Clinical College, Chongqing Medical University, Chongqing 400016, China; 4 College of Public Health, Chongqing Medical University, Chongqing 400016, China

**Keywords:** COVID-19, mental health, anxiety, depression, college student, risk factors

## Abstract

Background: Students are a priority population in mental health research. This study aimed to explore the risk factors of anxiety and depression symptoms among college students in Chongqing, a megacity under the impact of COVID-19, and to provide a basis for determining the priorities of public health policies and implementing effective educational health care interventions. Methods: In this cross-sectional study conducted in Chongqing, China, the data came from web-based stratified random sampling. Anxiety and depression symptoms were measured by the Self-Rating Anxiety Scale (SAS) and the Center for Epidemiological Studies Depression Scale (CES-D), respectively, and risk factors were analyzed by logistic regression. Results: Data were obtained from 915 college students (34.75% were male, and 65.25% were female) with age (20.29 ± 1.51) in Chongqing, China. The prevalence rates of anxiety and depression were 19.78% and 22.62%, respectively. Logistic regression analysis revealed that the risk factors of anxiety symptoms were associated with junior years, sleep time of less than 6 h a day, influence on career planning, and depression symptoms. Comprehensive, science and engineering, and medicine disciplines, having siblings, poorer mastery of study, and anxiety symptoms were risk factors for developing depression symptoms. Conclusions: During the pandemic, college students experienced varying degrees of anxiety and depression. Our research findings highlight the necessity of universities and relevant departments providing precise mental health education for college students under major public health emergencies.

## 1. Introduction

COVID-19 (coronavirus disease 19) has been shown to be a disease that predisposes people to mental debility [1,2]; clinical manifestations include mild symptoms such as headaches, loss of smell, loss of taste, fatigue, and other more serious illnesses [3]. Moreover, COVID-19 is also a major stressor that has led to a mental health crisis [4]. In the context of COVID-19 pandemic, some people report symptoms of sleep deprivation, depression, and anxiety [5]. A study [6] of different populations (including the general public, health workers, university students, older adults, infected patients, survivors of infection, and pregnant women) reported 23.1–43.3% pooled prevalence of depression in the population after the occurrence of COVID-19, 25.0–43.3% pooled prevalence of anxiety and 29.7–58.4% prevalence of insomnia, respectively. It is worth noting that the above 43.3% of the prevalence of depression symptoms and 58.4% of the population with insomnia symptoms were found in college students. Adequately, the growing trend of this adverse mental health problem is moving towards a younger age [7]. Therefore, it is crucial to comprehend the psychological well-being and risk factors among college students in the post-pandemic era, as this knowledge will serve as a valuable guide in assisting students with reintegrating into regular life and fostering a healthy mindset following the epidemic.

In the post-pandemic era, mental health has emerged as one of the most significant challenges since the outbreak of COVID-19 [8,9]. Previous studies [10,11] have indicated that this current generation of college students is particularly vulnerable to its impact. COVID-19 has affected their behavioral health across various domains, including academic pursuits, social interactions, daily routines, and career prospects, thereby exacerbating mental health issues. Additionally, there are many factors that can further contribute to the mental health status of college students during the pandemic, including anxiety and depression symptoms [12]. If these negative emotions are not able to be reasonably and effectively released in a timely manner, they might bring more troubles and obstacles to their studies and life.

Modern science regards anxiety symptoms as a mental state or a set of physiological reactions, which is a normal response to potential threats. The feeling of anxiety was a normal part of human experience, but excessive or inappropriate anxiety can become a disease [13,14]. Existing studies have shown that depression symptoms were a major contributor to the global burden of disease and a serious mental health problem [15]. At the same time, its prevalence increases significantly during adolescence and is prone to relapse in adulthood [16,17]. This type usually shows unstable changes in mood and psychological behavior [18]. Therefore, the prevention and control of anxiety and depression symptoms in young people are of great significance in reducing the occurrence of adverse mental health problems [19].

Studies in the USA [20] and France [21] indicated that the outbreak of COVID-19 has led to a high detection rate of anxiety (27.5%) and depression (16.1%) symptoms among college students. In addition, a cross-country study of nine countries including Poland, Russia, and Germany also confirmed this conclusion (anxiety: 30%; depression: 40.3%) [22]. At the same time, before the occurrence of COVID-19, the detection rates of anxiety and depression in the college students’ given group were only 12.94% and 19.53% [23]. Apparently, a large increase in such values has occurred, and a survey of adults in the Republic of Ireland by Philip Hyland et al. [24] similarly confirmed this growing trend. Current research indicates that the pandemic has had a significant impact on the mental health of college students, with an increase in the number of students experiencing negative emotions and psychological issues. This highlights the potential mental health consequences of COVID-19 on this population [25].

Most studies have focused on the developed areas of China [26], whereas Chongqing in Western China has rarely been reported. As one of the four municipalities directly under the Central Government of China and the leading city in Western China, Chongqing is a “megacity“ with 30 million people and nearly 1 million college students. This study can help us to better understand the mental health status of college students in the context of the COVID-19 pandemic in a megacity. This information will provide significant reference value for megacities worldwide, enabling them to develop effective intervention measures for the management and education of college students’ mental health after major public health emergencies. In addition, this study can also provide us with the latest data on the mental health issues of college students to better understand and solve the related problems.

## 2. Material and Methods

### 2.1. Study Design and Participants

This study was conducted in Chongqing, China from 18 January to 18 February 2022. During this period, the campus was under semi-closed management measures, and it was recommended that students do not leave the campus without permission. The questionnaires were distributed using web-based stratified random sampling (stratified by discipline type and grade) on social platforms (WeChat, QQ, Weibo, etc.), distributed on campus by investigators and quality control officers, and assisted by counselors of relevant universities. In this study, a total of 925 questionnaires were distributed to over 20 colleges in Chongqing, China, of which 915 met the inclusion and exclusion criteria, resulting in a questionnaire validity rate of 98.92%. Among them, the minor involved in the investigation has obtained the consent of his parents or guardians.

Inclusion criteria: (a) college students in Chongqing; (b) age between 16 and 30 years old.

Exclusion criteria: (a) questionnaires with identical answers to each item or a predetermined regularity option; (b) did not answer all questions; (c) students with confirmed COVID-19 infection.

### 2.2. Sample Size

The sample size of this study was estimated on the basis of the current situation survey formula $N=Z_{1-\alpha /2}^{2}\left(1-p\right)/\varepsilon^{2}p,\;\alpha =0.05,\;Z_{\alpha /2}^{\;}=1.96$. The prevalence of depression among college students during COVID-19 was about 34% [27], p=0.34, ε=0.1. The required sample size was calculated to be 746 people, and 10% efficiency is not expected, so the minimal sample size was 821.

### 2.3. Measures and Contents

The contents of the questionnaire covered demographics, awareness of COVID-19, the assessment of anxiety and depression symptoms.

#### 2.3.1. Demography

Self-compiled demographic characteristics questionnaire was used to collect information including the participants’ discipline, gender, grade, age, family circumstances, studies and life, and 2019 novel coronavirus vaccination status.

#### 2.3.2. Awareness of COVID-19

The issues in this section were intended to assess students’ awareness and attitude of various aspects of study and life as influenced by COVID-19, which was guided by the classical theory/model of health communication with respect to knowledge, attitudes, beliefs, and practices (KABP) [28], including the participants’ concern about COVID-19, the threat perception attitude toward COVID-19 (including concern for their families’ and their own safety during COVID-19, the cognitive attitude that COVID-19 will have serious impact and last for a long time), and the personal behavioral factors under the continuous epidemic (including personal opinion about the impacts of COVID-19 on study, life and career planning, emotion and the attitude towards regular epidemic prevention and control on campus, etc.).

#### 2.3.3. Anxiety

Anxiety symptoms were measured using the Zung’s Self-Rating Anxiety Scale (SAS) [29,30], which was created by William W.K. Zung in 1971 and is one of regularly used estimation apparatuses in clinical preliminaries for mental condition assessments in different fields, with wide pertinence and great inward consistency. The scale contains 20 items scored on a four-grade scale, and a total score of 50 points and above are considered to have anxiety symptoms, and the higher the standard score, the more severe the anxiety symptoms. The standardized Cronbach’s α coefficient for this study was 0.886.

#### 2.3.4. Depression

Depression symptoms were measured by the Center for Epidemiological Studies Depression Scale (CES-D) [31], which was created by Sirodff in 1977. The scale consists of 20 items with a total score of 60, and a score of 16 and above is considered to have depression symptoms, with higher scores indicating more pronounced depression symptoms. The normalized Cronbach’s α coefficient for this study was 0.897.

### 2.4. Quality Control and Ethical Considerations

The self-compiled questionnaire was finished through expert consultation, repeated discussion, and a pre-survey of 63 participants. This survey met the unified quality control requirements of the entire quality control process to ensure the reliability of the survey data. The Questionnaire Star (https://www.wjx.cn accessed on 18 January 2022) was used to collect data online using an anonymous approach. All participants signed electronic informed consent prior to registration. To avoid the same participant repeatedly answering the questionnaire, each device (e.g., mobile phone or computer) was eligible to answer only once, and logic checks were concurrently running in the background to identify invalid questionnaires. This study was approved by the Ethics Committee of Chongqing Medical University, Chongqing, China.

### 2.5. Statistical Analysis

Statistical analyses were performed with SAS version 9.2 (SAS Institute, Cary, NC, USA). In the statistical description of the data, the enumeration data were expressed as frequency and composition ratio (%), and the measurement data were expressed as mean and standard deviation $\bar{x}\pm s$. The software was also used to perform Spearman’s correlation analysis of the scores of each item of Zung’s Self-Rating Anxiety Scale (SAS) and the Center for Epidemiological Studies Depression Scale (CES-D). Univariate analysis of anxiety and depression was performed using the χ^2^ test and Fisher’s exact test. Bonferroni correction was used for multiple tests. The OR (odds ratio) values and 95% CI (confidence interval) were calculated using logistic regression at the test-level of α = 0.05.

## 3. Results

### 3.1. Participants’ Characteristics

Among the 915 participants, there were 318 males and 597 females with a mean age of 20.29 years (range 16–30 years). In addition, among their discipline types, 20.77% (190) were from comprehensive colleges, 28.42% (260) were students studying science and engineering, 23.28% (213) were students studying literature, and 27.54% (252) were students studying medicine. Simultaneously, 461 (50.38%) participants had no siblings. The majority of participants were urban dwellers (54.43%), and most were vaccinated throughout (88.31%) (Table 1).

### 3.2. The Prevalence of Anxiety and Depression

The anxiety and depression self-assessment scales of students in Chongqing were scored (41.89 ± 9.83) and (11.20 ± 7.99), respectively, and the scores were correlated significantly (ρ = 0.53, *p* < 0.01). The detection rates of anxiety and depression symptoms were 19.78% (181/915) and 22.62% (207/915). Among them, 101 (11.04%) participants had both anxiety and depression symptoms (Table 2).

### 3.3. Univariate Analysis of Anxiety and Depression

The univariate analysis of anxiety symptoms showed that among the factors of study and life, there was a statistically significant difference in daily sleep time (*p* < 0.01). Among the factors of cognitive factors under the context of COVID-19, there was a statistically significant difference (*p* < 0.05) in the attitude toward COVID-19 will last for a long time. Among the personal behavior factors under the context of COVID-19, personal opinion about the impact of the ongoing epidemic situation on the life, career planning, and mood swings differed significantly between groups (all *p* < 0.05). The group distribution of the remaining variables was not statistically significant. In the subsequent Bonferroni multiple comparison test, the variable “Personal opinion about the impact of COVID-19 on life” was excluded.

The univariate analysis of depression symptoms showed that among the social demographic factors, there were statistically significant differences in the different discipline types (*p* < 0.01). Among the family factors, place of residence (*p* < 0.01), being the only child or not (*p* < 0.01), and living with parents (*p* < 0.05) showed statistically significant variations. Among the factors of study and life, there were statistically significant differences in study mastery, frequency of exercise, and daily sleep time (all *p* < 0.01). Among the cognitive factors under the context of COVID-19, the differences in knowledge about COVID-19 were statistically significant (*p* < 0.01). Among the personal behavioral factors, the differences in the acceptance of online teaching, personal opinion about the impact of COVID-19 on study and life, and attitudes toward regular epidemic prevention and control on campus were statistically significant (all *p* < 0.05) (Table 3). Among the above variables, all variables passed the multiple tests of Bonferroni correction.

### 3.4. Multivariate Analysis of Anxiety and Depression

In the multivariate analysis of anxiety and depression, whether the participants suffered from anxiety (0 = no, 1 = yes) or depression (0 = no, 1 = yes) symptoms were used as dependent variables. The difference in univariate analysis was statistically significant (*p* < 0.05), and the variables that passed the multiple comparison test of Bonferroni correction were used as independent variables for logistic regression analysis. The spearman correlation analysis showed that there was a correlation between whether they suffered from anxiety and depression (*p* < 0.05), so each of them was included as an independent variable in the multifactorial analysis of depression and anxiety, respectively.

#### 3.4.1. Multivariate Analysis of Anxiety

Logistic regression analysis revealed that the factors influencing anxiety symptoms were the grade, daily sleep time, personal opinion about the impact of COVID-19 on career planning, and whether they suffered from depression symptoms (*p* < 0.05).

Compared with freshmen, the risk of suffering from anxiety symptoms was 2.29 times greater in junior college students (OR = 2.29, 95% CI: 1.27, 4.15). Students whose daily sleep time was less than 6 h had a higher risk of suffering from anxiety symptoms compared to those who slept 8–10 h (excluding 10 h) a day (OR = 4.44, 95% CI: 2.04, 9.66). At the same time, it was believed that COVID-19 had a greater impact on career planning and was also a risk factor for anxiety symptoms in college students (OR = 2.34, 95% CI: 1.11, 4.96). Likewise, students within depression symptoms were more likely to have anxiety symptoms (OR = 7.69, 95% CI: 5.28, 11.21) (Table 4).

#### 3.4.2. Multivariate Analysis of Depression

Logistic regression analysis showed that the factors influencing depression symptoms were discipline types, being the only child or not, study mastery, attitudes toward regular epidemic prevention and control measures on campus, and whether they suffered from anxiety symptoms (*p* < 0.05).

The discipline types of the participants that included comprehensive studies, science and engineering, and medicine were exposure factors for suffering from depression symptoms compared to the literature discipline (OR = 4.48, 95% CI: 2.30, 8.73; OR = 3.17, 95% CI: 1.70, 5.93; OR = 5.87, 95% CI: 3.16, 10.91, respectively). Compared to being the only child, having siblings was 1.70 times more likely to have depression symptoms (OR = 1.70, 95% CI: 1.17, 2.48). Moderate and poor mastery of the study were exposure factors for developing depression symptoms more than good mastery of the study (OR = 1.81, 95% CI: 1.25, 2.62; OR = 4.33, 95% CI:1.18, 15.84). Moreover, moderate acceptance and nonacceptance of regular epidemic prevention and control measures on campus compared to full acceptance were exposure factors for developing depression symptoms (OR = 1.80, 95% CI: 1.04, 3.11; OR = 3.41, 95% CI: 1.58, 7.36). Additionally, having anxiety symptoms would more likely lead to depression (OR = 9.42, 95% CI: 6.29, 14.12) (Table 5).

## 4. Discussion

### 4.1. Prevalence of Anxiety and Depression

The cross-sectional survey among college students in Chongqing during the post-pandemic era under the impact of COVID-19 showed that anxiety and depression were common among college students. The detection rates of anxiety and depression symptoms in this study were 19.78% and 22.62%, which were higher than 12.94% and 19.53% of anxiety and depression in college students before COVID-19 [23]. This fact has been confirmed by a number of similar studies of young people in different regions during this period, including the United States, Britain, Italy, and China, to name but a few [32,33,34]. One of the studies [32] on young people in Vermont in the United States pointed out that the prevalence of anxiety and depression symptoms after COVID-19 was 1.34 and 1.31 times higher than before, which is a noteworthy growth trend. It can be seen that in this context, the detection rate of anxiety and depression symptoms of college students shows an upward trend. Related observational studies have also shown that the population was prone to anxiety and depression in the context of COVID-19 [35,36]. Such situations require support for effective response practices and the provision of effective social resources [37].

With the rapid development of global and Chinese epidemic prevention and control strategies in the past three years, the initial panic and fear of COVID-19 have eased slightly. However, young people may be particularly vulnerable to the consequences of mental health in this context [38], so its long-term impact on the growth and development of college students deserves special attention [39]. At the same time, the study found that the detection rate of anxiety symptoms was 24.23% higher in science and engineering students, but the difference was not statistically significant. The detection rate of depression in Chinese medicine students was 32.14%, which was the highest, and the difference was statistically significant. This may be caused by the greater learning pressure, higher professional requirements and greater employment competition of students in these two majors. In addition, a correlation and interactions between anxiety and depression symptoms were also found in this study, which is similar to some research results [40,41,42]. Therefore, we should focus on the mental health of groups with comorbidity of anxiety and depression symptoms and provide precise intervention when necessary to help them to recover to a healthy level. After a major public health event, the number of people affected by mental health was often greater than the number of physical injuries, and the impact of mental health may last longer [43]. In general, the results of our study showed that anxiety and depression symptoms were common among college students in Chongqing, and that the persistent impact of COVID-19 on mental health deserves focused attention.

### 4.2. Risk Factors of Anxiety

Our study found that risk factors for anxiety included the junior year, daily sleep time of less than 6 h, personal opinion that COVID-19 had more influence on career planning, and suffering from depression symptoms.

Compared with freshmen, the anxiety symptoms of junior college students were particularly obvious, with grade differences. Upperclassmen college students become more susceptible to mental health issues due to the epidemic [44], which in turn were prone to anxiety symptoms [45]. During the third school year, students are faced with the choice of taking the postgraduate entrance examination or finding a job, as well as academic pressure, which may make their emotions fluctuate more and the students themselves prone to negative emotions [46]. Public health emergencies can lead to a difficult current employment situation and increased pressure on employment [47], which further increases the concern of fresh graduates about the unknown situation [48], making anxiety symptoms appear.

For career planning, students who thought that COVID-19 had a greater impact on their future career planning were also more likely to have anxiety symptoms compared to those who thought that it had a low impact. In the context of COVID-19, college students were prone to poor mental health and experiencing pressure with respect to future careers [49]. For students to be employed after graduation, they should seize the opportunity to improve their abilities and make themselves active in the job market [50]. However, existing research shows that positive coping style can alleviate the negative emotions of college students to a large extent [51]. College students should be optimistic, actively adapt to the development of and changes in the epidemic and adjust their future plans and expectations accordingly to the current situation. Therefore, we believe that targeted attention should be paid to college students who think that COVID-19 has a greater impact on career planning, and psychological assistance should be provided to them when necessary, which will help to reduce the appearance of anxiety symptoms.

More importantly, we found that insufficient sleep time may lead to anxiety symptoms; the shorter the sleep time, the higher the risk of suffering from anxiety symptoms [52]. In our survey, most college students (67.21%) could ensure adequate sleep time every day, but 4.92% had a serious shortage in sleep time (less than 6 h). It is worth noting that sleeping less than 6 h a day was a strong risk factor for anxiety in college students, and the risk of anxiety was 4.44 times higher than that of sleeping 8–10 h (excluding 10 h) per day. Insufficient daily sleep duration is not only harmful to the body but also one of the reasons for inducing mental health problems [53]. Especially in the context of the normalization control of COVID-19, young people have poor sleep quality, and the detection rate of anxiety caused by sleep problems is high [53,54]. In particular, we found that under the impact of COVID-19, 8–10 h (excluding 10 h) of sleep time per day is the most recommended for college students, and that sleeping less than 6 h is dangerous. A focused intervention for college students who sleep less than 6 h a day can help them to develop a good routine and healthy psychological state [55].

### 4.3. Risk Factors of Depression

Our study revealed that the risk factors for depression included being students of comprehensive colleges, studying science and engineering and medicine, having siblings, lack of good study mastery, lower acceptance of epidemic prevention and control measures on campus, and having anxiety symptoms.

In terms of discipline types, medical students had the highest risk of depression, up to 32.14%, compared to the students of comprehensive colleges and students studying science and engineering and literature. The reason may be that medical students have greater academic pressure, which makes students prone to mental health problems [56]. Medical courses are usually professional and difficult to understand, and excessive learning pressure can lead to negative emotions [57]. In addition, medical students know more about the disease and are more likely to pay attention to the serious consequences of the disease [58]. To a certain extent, there is a gap in understanding among students of other disciplines [59]. In the context of COVID-19, it has also increased medical students’ concerns about their health status and thus their tendency to show depression [60]. More importantly, the protection of and attention to the mental health of medical students are conducive to improving the quality of future doctors and promoting the development of medicine [61]. Therefore, medical colleges should express more concern for the mental health of their students, create a good atmosphere for learning and living, and promote a more favorable development of students’ mental health.

Regarding the family situation, compared with the only-child students, college students having siblings were 1.70 times more likely to suffer from depression, which may be related to the concept of family education and parenting style [62]. In daily life, the only child may always receive fuller and continuous care from the parents. Effective interaction and communication will make their mental state more stable [63]. However, college students having siblings often need to consider their siblings’ feelings and compete with their siblings for their parents’ attention [64]. Learning and life full of comparisons may damage their self-confidence, so that they may be prone to experiencing certain psychological pressures, and this growth period is often critical for the emergence and deterioration of adverse mental health symptoms [38]. Therefore, our suggests that families with two or more children should pay more attention to their mental health.

In the matter of study mastery, college students who had good mastery of their studies were significantly less likely to suffer from depression symptoms compared to students with moderate and poor mastery of learning. Studying is the main task of college students at this stage. At the same time, there is a direct relationship between the mastery of course studies and academic performance, and poorer mastery of studies may lead to an increase in their psychological burden, which is also closely related to depression [65]. Previous studies indicate that students who have a good grasp of the learning courses have a better self-learning ability, can follow the developments of COVID-19 on time, and respond to the changes in the epidemic with positive behavior [66]. It has also been shown that people who cope well with the epidemic are less likely to develop psychological disorders such as depression [67].

In addition, our study revealed that the risk of depression was 3.41 times higher among students who were not accepting the epidemic prevention and control measures on campus compared to those who were accepting them. A longitudinal study also showed an increase in depression symptoms in the two months following the imposition of restrictions [62]. Long time in a relatively closed environment to study and live, resulting in reduced contact with the outside world. At the same time, it lacks the stimulation of new things from the outside world, which gradually leads to fatigue and reduction in positive emotions [68]. Compared to other adults, college students do not have enough social and life experience and are more vulnerable to negative emotions, which increases the risk of depression [69], and may even harm their study [70]. This period of growth is a key stage in the development and maintenance of mental health towards independent adulthood [71]. Therefore, under this condition, colleges should perform multilevel comprehensive management concerning students’ studies and daily life, and actively focus on mental health education and early identification of and intervention into psychological crises.

To our knowledge, this study is the first to investigate the mental health status of college students in Chongqing in the post-pandemic era. Therefore, it provides valuable insights into the mental health status of local college students. Our results show that in the post-epidemic era, many factors discussed in this study can directly affect the mental health symptoms of college students, thereby inducing the occurrence of anxiety and depression symptoms. In order to keep college students in good mental health, we should advocate that they get enough sleep in addition to regular physical exercise [44]. Sufficient aerobic and muscle-strengthening exercises can effectively alleviate bad moods or reduce the occurrence of poor mental health symptoms, thus strengthening our resilience to stress [72]. At the same time, relevant departments and colleges should also be encouraged to provide more precise interventions and mental health education for college students. The benefits of universal resilience-focused interventions for specific populations lie in the fact that they are most likely to reduce anxiety and depression symptoms in the short term [73,74].

### 4.4. Limitations

It is important to note several limitations in our study. Firstly, a cross-sectional analysis was adopted for this study, which cannot determine the causal relationship between the variables and psychological changes in the participants before and after the epidemic. For this, we considered further exploration through a cohort study to continue to focus on clinical psychological changes in the participants. Secondly, this study utilized a self-report method for data collection, which may be subject to the influence of individual subjective responses in data reporting. We used a scale evaluation to assess the mental health status of the subjects, but no clinical diagnosis was made. In addition, we plan to increase the sample size in future studies to explore the risk factors for anxiety and depression symptoms and their associations.

## 5. Conclusions

This study found that the impact of COVID-19 has resulted in a high detection rate of anxiety and depression symptoms among college students in Chongqing, China. Focused attention should be paid to the mental health status of college students who are seniors, study medicine, have siblings, sleep less than 6 h a day, and have poor mastery of their studies. In our study, it was found that ensuring 8–10 h (excluding 10 h) of adequate sleep every day had a positive effect on the healthy psychological status of college students in the context of COVID-19. At the same time, students who had more knowledge about the epidemic were less likely to experience anxiety and depression symptoms. In this regard, relevant government departments and universities can carry out targeted mental health education activities to guide students to actively respond to the normalized development of the epidemic prevention and control measures. In addition, it is essential to pay attention to school mental health education under the campus normalization epidemic prevention and control measures, and to strengthen the guidance of students in study and career planning.

## Figures and Tables

**Table 1:** Demographics of college students in Chongqing (*N* = 915).

Characteristics	*N*(Mean)	%(SD)	Characteristics	*N*(Mean)	%(SD)
Gender			Discipline types		
Male	318	34.75	Literature	213	23.28
Female	597	65.25	Comprehensive	190	20.77
Age ^#^	20.29	1.51	Science and engineering	260	28.42
<18	4	0.44	Medicine	252	27.54
18–22	860	93.99	Only child		
>22	51	5.57	Yes	461	50.38
Place of residence			No	454	49.62
Urban	498	54.43	Living with parents		
Rural	417	45.57	Live with both parents	669	73.11
Grade			Live with one parent	172	18.80
Freshman	193	21.09	Living without parents	74	8.09
Sophomore	267	29.18	2019 novel coronavirus vaccination status		
Junior	163	17.81	All	808	88.31
Senior and above	292	31.91	Part	105	11.48
			None	2	0.22

The superscript with “^#^” was described using the mean and standard deviation, and the rest was described using the frequency and composition ratio. Abbreviations: SD, standard deviation.

**Table 2:** The prevalence and correlation of anxiety and depression among college students in Chongqing.

	Prevalence		Scores		Correlation		Complication	
	n	%	Mean	Std	ρ	*p*	n	%
Anxiety	181	19.78	41.89	9.83	0.53	<0.01 **	101	11.04
Depression	207	22.62	11.20	7.99				

** refers to *p* < 0.01. Abbreviations: Std: standard.

**Table 3:** Univariate analysis of anxiety and depression of college students in Chongqing.

Characteristics	Total		Anxiety				Depression			
	n	%	n	%	*x* ^2^	*p*	n	%	*x* ^2^	*p*
*N*	915	100.00	181	19.78			207	22.62		
**Social Demographic Factors**										
Discipline types										
Literature	213	23.28	42	19.72	5.89	0.117	19	8.92	36.41	<0.001 **
Comprehensive	190	20.77	29	15.26			47	24.74		
Science and engineering	260	28.42	63	24.23			60	23.08		
Medicine	252	27.54	47	18.65			81	32.14		
Gender										
Male	318	34.75	60	18.87	0.26	0.613	72	22.64	0.00	0.992
Female	597	65.25	121	20.27			135	22.61		
Grade										
Freshman	193	21.09	31	16.06	3.09	0.378	49	25.39	7.38	0.061
Sophomore	267	29.18	55	20.60			71	26.59		
Junior	163	17.81	38	23.31			27	16.56		
Senior and above	292	31.91	57	19.52			60	20.55		
Age										
<18	4	0.44	1	25.00		0.826 ^#^	1	25.00		0.604 ^#^
18–22	860	93.99	171	19.88			197	22.91		
>22	51	5.57	9	17.65			9	17.65		
**Family Factors**										
Place of residence										
Urban	498	54.43	94	18.88	0.57	0.452	94	18.88	8.77	0.003 **
Rural	417	45.57	87	20.86			113	27.10		
Only child										
Yes	461	50.38	85	18.44	1.06	0.304	77	16.70	18.60	<0.001 **
No	454	49.62	96	21.15			130	28.63		
Living with parents										
Live with both parents	669	73.11	128	19.13	2.66	0.264	145	21.67	7.24	0.027 *
Live with one parent	172	18.80	33	19.19			36	20.93		
Living without parents	74	8.09	20	27.03			26	35.14		
**Study and Life Factors**										
Study mastery										
Good	559	61.09	103	18.43	2.33	0.312	98	17.53	25.44	<0.001 **
Moderate	343	37.49	74	21.57			102	29.74		
Poor	13	1.42	4	30.77			7	53.85		
Frequency of exercise										
Daily	65	7.10	15	23.08	0.79	0.940	17	26.15	14.38	0.006 **
5–6 times a week	84	9.18	16	19.05			9	10.71		
3–4 times a week	261	28.52	49	18.77			49	18.77		
1–2 times a week	421	46.01	83	19.71			106	25.18		
Never	84	9.18	18	21.43			26	30.95		
Daily sleep time										
<6 h	45	4.92	23	51.11	30.33	<0.001 **	19	42.22	13.63	0.004 **
6–8 h (excluding 8 h)	615	67.21	116	18.86			143	23.25		
8–10 h (excluding 10 h)	237	25.90	38	16.03			42	17.72		
≥10 h	18	1.97	4	22.22			3	16.67		
**Cognitive Factors under the Context of COVID-19**										
2019 novel coronavirus vaccination status										
All	808	88.31	160	19.80		0.513 ^#^	180	22.28		0.397 ^#^
Part	105	11.48	20	19.05			26	24.76		
None	2	0.22	1	50.00			1	50.00		
Knowledge about COVID-19										
Complete	167	18.25	32	19.16	1.76	0.623	26	15.57	12.14	0.007 **
Considerable	513	56.07	104	20.27			123	23.98		
Moderate	219	23.93	40	18.26			50	22.83		
Poor	16	1.75	5	31.25			8	50.00		
Not at all	0	0.00	0	0.00			0	0.00		
Concern about COVID-19										
Quite concerned	195	21.31	39	20.00	1.89	0.595	37	18.97	5.62	0.132
Comparatively concerned	483	52.79	102	21.12			112	23.19		
Moderately concerned	205	22.40	34	16.59			46	22.44		
Less concerned	32	3.50	6	18.75			12	37.50		
Not concerned	0	0.00	0	0.00			0	0.00		
Worried about the safety of yourself and family										
Fully conform	199	21.75	38	19.10	3.68	0.451	47	23.62	0.77	0.942
Relatively consistent	305	33.33	67	21.97			72	23.61		
Moderately	289	31.58	59	20.42			62	21.45		
Comparatively not	71	7.76	10	14.08			16	22.54		
Not at all	51	5.57	7	13.73			10	19.61		
COVID-19 brings a serious impact										
Fully conform	288	31.48	54	18.75	1.17	0.883	61	21.18	4.12	0.390
Relatively consistent	300	32.79	63	21.00			73	24.33		
Moderately	245	26.78	49	20.00			59	24.08		
Comparatively not	65	7.10	13	20.00			13	20.00		
Not at all	17	1.86	2	11.76			1	5.88		
COVID-19 will last for a long time										
Fully conform	213	23.28	57	26.76	12.75	0.013 *	54	25.35	7.60	0.108
Relatively consistent	314	34.32	57	18.15			68	21.66		
Moderately	299	32.68	54	18.06			70	23.41		
Comparatively not	59	6.45	12	20.34			14	23.73		
Not at all	30	3.28	1	3.33			1	3.33		
**Personal Behavior Factors under the Context of COVID-19**										
Acceptance of online teaching										
Full acceptance	582	63.61	112	19.24	0.30	0.860	121	20.79	8.45	0.015 *
Moderate acceptance	264	28.85	55	20.83			61	23.11		
Nonacceptance	69	7.54	14	20.29			25	36.23		
Personal opinion about the impact of COVID-19 on study										
Greater impact	508	55.52	114	22.44	5.73	0.057	131	25.79	8.02	0.018 *
Moderate impact	351	38.36	60	17.09			62	17.66		
Low impact	56	6.12	7	12.50			14	25.00		
Personal opinion about the impact of COVID-19 on life										
Greater impact	578	63.17	123	21.28	6.01	0.050 *	151	26.12	11.00	0.004 **
Moderate impact	308	33.66	57	18.51			51	16.56		
Low impact	29	3.17	1	3.45			5	17.24		
Personal opinion about the impact of COVID-19 on career planning										
Greater impact	485	53.01	114	23.51	9.91	0.007 **	119	24.54	3.02	0.221
Moderate impact	346	37.81	57	16.47			74	21.39		
Low impact	84	9.18	10	11.90			14	16.67		
Attitudes toward regular epidemic prevention and control measures on campus										
Full acceptance	138	15.08	27	19.57	2.60	0.272	24	17.39	12.45	0.002 **
Moderate acceptance	707	77.27	135	19.09			156	22.07		
Nonacceptance	70	7.65	19	27.14			27	38.57		
Personal opinion about the impact of COVID-19 on mood swings										
Greater impact	282	30.82	68	24.11	9.02	0.011 *	70	24.82	2.23	0.328
Moderate impact	477	52.13	94	19.71			108	22.64		
Low impact	156	17.05	19	12.18			29	18.59		

The superscript with “^#^” indicates the use of Fisher’s exact probability test, and the rest were tested by the chi-square test; * refers to *p* < 0.05, and ** refers to *p* < 0.01.

**Table 4:** Logistic regression analysis of anxiety in college students in Chongqing.

Variables	B	p-Value	OR	95% CI for OR		Forest Map
				Lower	Upper	
Intercept	−3.18	<0.001				
Grade						
Freshman (ref)						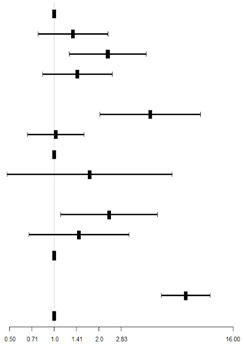
Sophomore	0.29	0.289	1.34	0.78	2.30	
Junior	0.83	0.006 **	2.29	1.27	4.15	
Senior and above	0.36	0.193	1.43	0.83	2.46	
Daily sleep time						
<6 h	1.49	<0.001 **	4.44	2.04	9.66	
6–8 h (excluding 8 h)	0.03	0.906	1.03	0.66	1.59	
8–10 h (excluding 10 h) (ref)						
≥10 h	0.55	0.399	1.73	0.48	6.23	
Personal opinion about the impact of COVID-19 on career planning						
Greater impact	0.85	0.026 *	2.34	1.11	4.96	
Moderate impact	0.39	0.330	1.47	0.68	3.19	
Low impact (ref)						
Depression						
Yes	2.04	<0.001 **	7.69	5.28	11.21	
No (ref)						

* refers to *p* < 0.05, ** refers to *p* < 0.01. Abbreviations: ref, reference level; CI, confidence interval.

**Table 5:** Logistic regression analysis of depression of college students in Chongqing.

Variables	B	p-Value	OR	95% CI for OR		Forest Map
				Lower	Upper	
Intercept	−4.20	<0.001				
Discipline types						
Literature (ref)						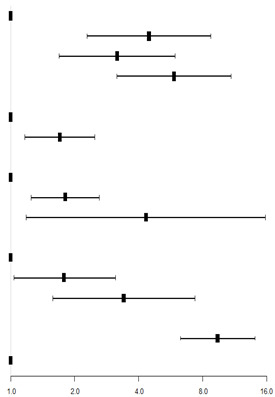
Comprehensive	1.50	<0.001 **	4.48	2.30	8.73	
Science and engineering	1.15	<0.001 **	3.17	1.70	5.93	
Medicine	1.77	<0.001 **	5.87	3.16	10.91	
Only child						
Yes (ref)						
No	0.53	0.006 **	1.70	1.17	2.48	
Study mastery						
Good (ref)						
Moderate	0.59	0.002 **	1.81	1.25	2.62	
Poor	1.47	0.027 *	4.33	1.18	15.84	
Attitudes toward regular epidemic prevention and control measures on campus						
Full acceptance						
Moderate acceptance	0.59	0.037 *	1.80	1.04	3.11	
Nonacceptance	1.23	0.002 **	3.41	1.58	7.36	
Anxiety						
Yes	2.24	<0.001 **	9.42	6.29	14.12	
No (ref)						

* refers to *p* < 0.05, ** refers to *p* < 0.01. Abbreviations: ref, reference level; CI, confidence interval.

## Data Availability

The data underlying this article cannot be shared publicly due to the privacy of individuals that participated in this study and will be shared on reasonable request to the corresponding author.
